# When brain and heart collide: a deeper dive into treatment pathways of stroke complicating TAVI

**DOI:** 10.1007/s12928-025-01121-w

**Published:** 2025-03-29

**Authors:** Mark Kheifets, Boris Kruchin, Guy Witberg, Tsahi T. Lerman, Rani Barnea, Michael Findler, Ran Brauner, Leor Perl, Pablo Codner, Yeela Talmor-Barkan, Guy Rephaeli, Eitan Auriel, Ran Kornowski, Amos Levi

**Affiliations:** 1 Department of Cardiology, Rabin Medical Center, Petah Tikva, Israel; 2 Affiliated to the Faculty of Medicine, Tel Aviv University, Tel Aviv, Israel; 3 Department of Neurology, Rabin Medical Center, Petach Tikva, Israel; 4 Peter Munk Cardiac Centre, Toronto General Hospital, University Health Network, Toronto, Canada

**Keywords:** TAVI, AISCT, Neurointervention

## Abstract

Periprocedural ischemic stroke remains a major concern of transcatheter aortic valve implantation (TAVI). The aims of this study were to describe the incidence, characteristic, and outcomes of patients presenting with acute ischemic stroke complicating TAVI (AISCT), and to compare conservative management (CM) vs. neurointervention (NI), in eligible patients. We analyzed consecutive TAVI procedures performed in the Rabin Medical Center between 2008 and 2021. The patients with and without AISCT were compared. Outcomes of NI following AISCT were compared to CM. The primary outcomes were mortality at 1 and 3 years and neurologic disability status at 3 months. Of 1515 eligible patients who underwent TAVI between 2008 and 2021, 38 (2.5%) had AISCT within 30 days. All-cause mortality was significantly higher in the AISCT group in 30 days (13.9% vs. 2.4%, *p* < 0.01), 1 year (27.8% vs. 8.1%, *p* < 0.01), and 3 years (49.0% vs. 26.8%, *p* < 0.01), as compared to the AISCT negative group. Out of the 38 patients who suffered AISCT between 2008 and 2021, 7 underwent NI. The majority of AISCT were observed within the first 24-h following TAVI (*p* < 0.01). All-cause mortality following moderate/severe stroke was significantly higher (*p* = 0.037) compared to mild stroke. As compared to the CM, NI was not found to reduce mortality. However, patients with moderate severity stroke who underwent NI enjoyed a higher rate of disability free survival at 3 months (100% vs. 40%, *p* = 0.044), compared to patients treated conservatively. NI, as compared to CM, may improve disability status in eligible patients with moderate stroke.

**Graphical abstract:**

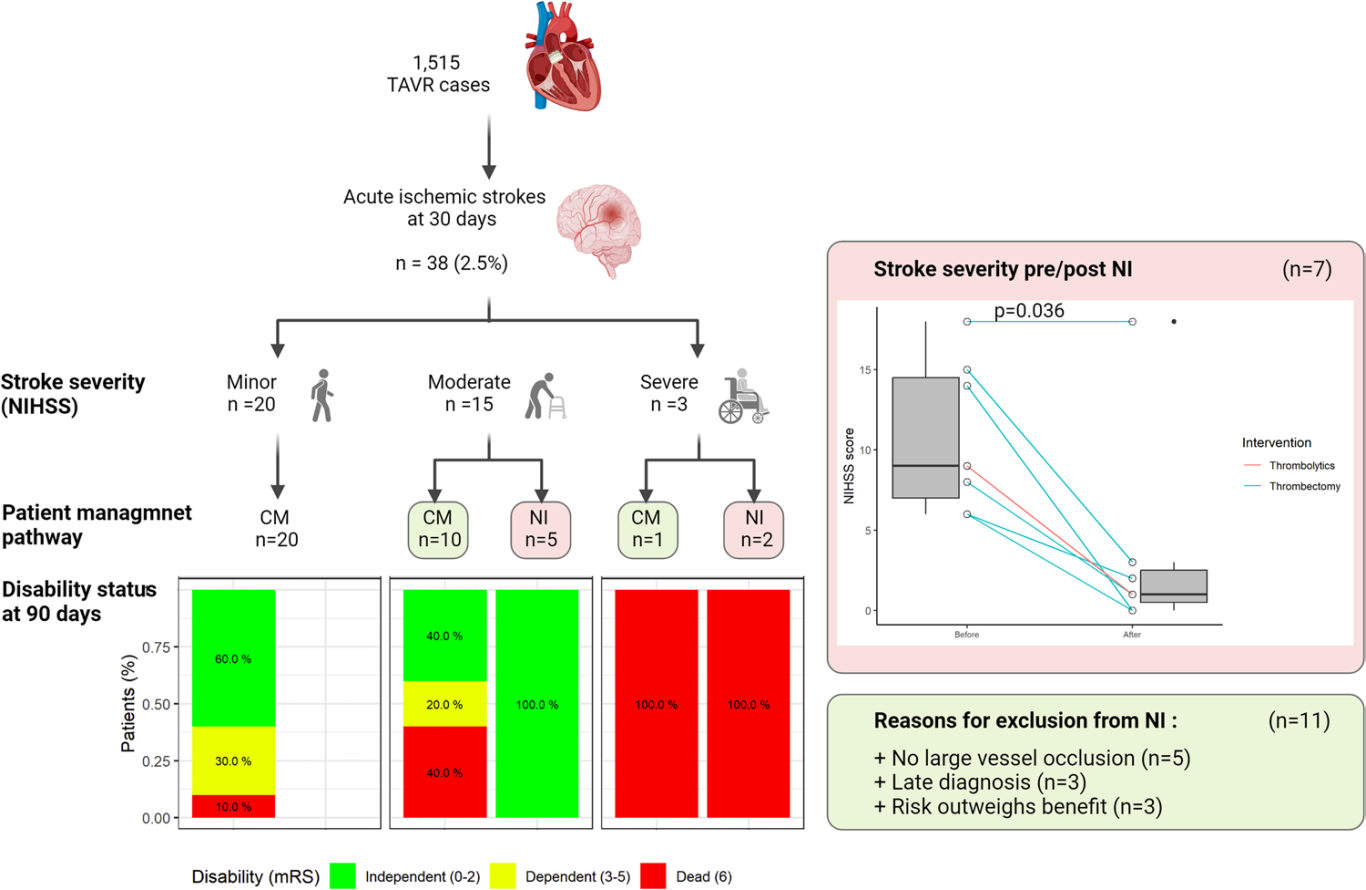

**Supplementary Information:**

The online version contains supplementary material available at 10.1007/s12928-025-01121-w.

## Introduction

Severe aortic valve stenosis is the most common primary valve disease requiring intervention in the developed world, with rapidly rising prevalence owing to population aging [1]. Since the first transcatheter aortic valve implantation (TAVI) preformed in 2002, TAVI has continued to expand across the surgical risk spectrum as a treatment option for patients with severe aortic stenosis (AS)—from its’ early days as a “last resort” for inoperable patients, to its current widespread use, including in patients with low surgical risk [2]. As a result, the annual volume of TAVI has increased every year since 2011, and in 2019, TAVI volume (*n* = 72,991) exceeded all forms of surgical aortic valve replacement (*n* = 57,626) in the United States. Subsequently, the decline in mortality from the early TAVI experience to 2019 has been steady and dramatic, with in-hospital mortality falling from 5.4 to 1.3%, and 30-day mortality decreasing from 7.2 to 2.5%. Accordingly, the srates of permanent pacemaker implantations, vascular complications, moderate to severe paravalvular leaks, major bleeding events, and need for renal replacement therapy have also declined. Notwithstanding, both the rate of in-hospital and 30-day stroke has remained high (1.6% and 2.3%, respectively), and stable [3]. This observation is unsettling, given that acute ischemic stroke complicating TAVI (AISCT) is associated with longer stay in the intensive care unit, longer overall hospitalization duration, lower quality of life (QOL) indices, and eventually higher mortality [4]. Parallel to the evolution of TAVI, much progress has been made in the fields of both prediction [5] and therapy [6] of AISCT cases. Nevertheless, studies describing patients who would benefit from neurointervention (NI) are scarce. Hence, the aim of our research was to describe the incidence and characteristics of patients with AISCT, specifically those who could benefit from early NI.

### Methods

We analyzed data from the Rabin medical center institutional TAVI registry (2008–2021).

The Valve Academic Research Consortium 3 (VARC-3) definitions were used to define and categorize neurological events [7]. Ischemic stroke was defined as acute onset of focal neurological signs or symptoms conforming to a focal or multifocal vascular territory within the brain, spinal cord, or retina (NeuroARC Type 1a or 1aH) and fulfilling one of the following criteria: (1) Signs or symptoms lasting ≥ 24 h or until death, with pathology or neuroimaging evidence of central nervous system (CNS) infarction, or absence of other apparent causes; (2) Symptoms lasting < 24 h, with pathology or neuroimaging confirmation of CNS infarction in the corresponding vascular territory. Hemorrhagic stroke was defined as acute onset of neurological signs or symptoms due to intraparenchymal, intraventricular, spinal cord, or retinal collection of blood (Symptomatic intracerebral hemorrhage [ICH], NeuroARC type 1b/ECASS parenchymal hematoma type II), or subarachnoid hemorrhage (Symptomatic subarachnoid hemorrhage [SAH], NeuroARC type 1c), not caused by trauma. Stroke not otherwise specified was defined as acute onset of neurological signs or symptoms persisting ≥ 24 h or until death but without sufficient neuroimaging or pathology evidence to be classified (NeuroARC Type 1d). Transient ischemic attack (TIA) was defined as transient focal neurological signs or symptoms lasting < 24 h presumed to be due to focal brain, spinal cord, or retinal ischemia, but without evidence of acute infarction by neuroimaging or pathology, or with no imaging performed (NeuroARC Type 3a or Type 3aH). Stroke severity was assessed using the National Institutes of Health Stroke Scale (NIHSS), with an NIHSS of 0–5 considered to be a mild stroke, 6–14 moderate, and ≥ 15 severe [8]. The primary outcomes were 1 and 3 years all cause death, and neurologic disability status at 90 days according to the modified Rankin score (mRS). Stroke disability status was classified as dead (mRS 6), alive but dependent (mRS 3–5 and increase of at least 1 from baseline) or alive and independent (mRS < 3 or without increase from baseline) [7]. The baseline characteristics were compared between the AISCT- and ACST + groups, and in the conservative management (CM) and NI groups. The continuous variables are reported as means (± SD), or medians and interquartile ranges, as appropriate. Categorical variables are described as percentages. The characteristics of study participants were compared using the Fischer test for categorical variables and Student’s *t*-test or Wilcoxon rank tests, as appropriate for continuous variables. All tests were 2-sided and a value of *p* < 0.05 was considered significant. Treatment modality dependent outcomes were compared in patients with moderate or severe stroke, considering that NI was almost exclusively reserved to treat moderate or severe stroke. Survival of the AISCT- vs AISCT + groups and the CM vs. NI groups is graphically displayed by Kaplan–Meier curves and compared using the log rank test (unadjusted analysis). Cox proportional hazards models were used to assess the impact of stroke severity on survival. Logistic regression models were used to examine possible predictors of AISCCT. All analyses were performed using R (R-studio, V.4.0.0, Vienna, Austria).

## Results

### Patient and procedural characteristics

**Fig. 1:**
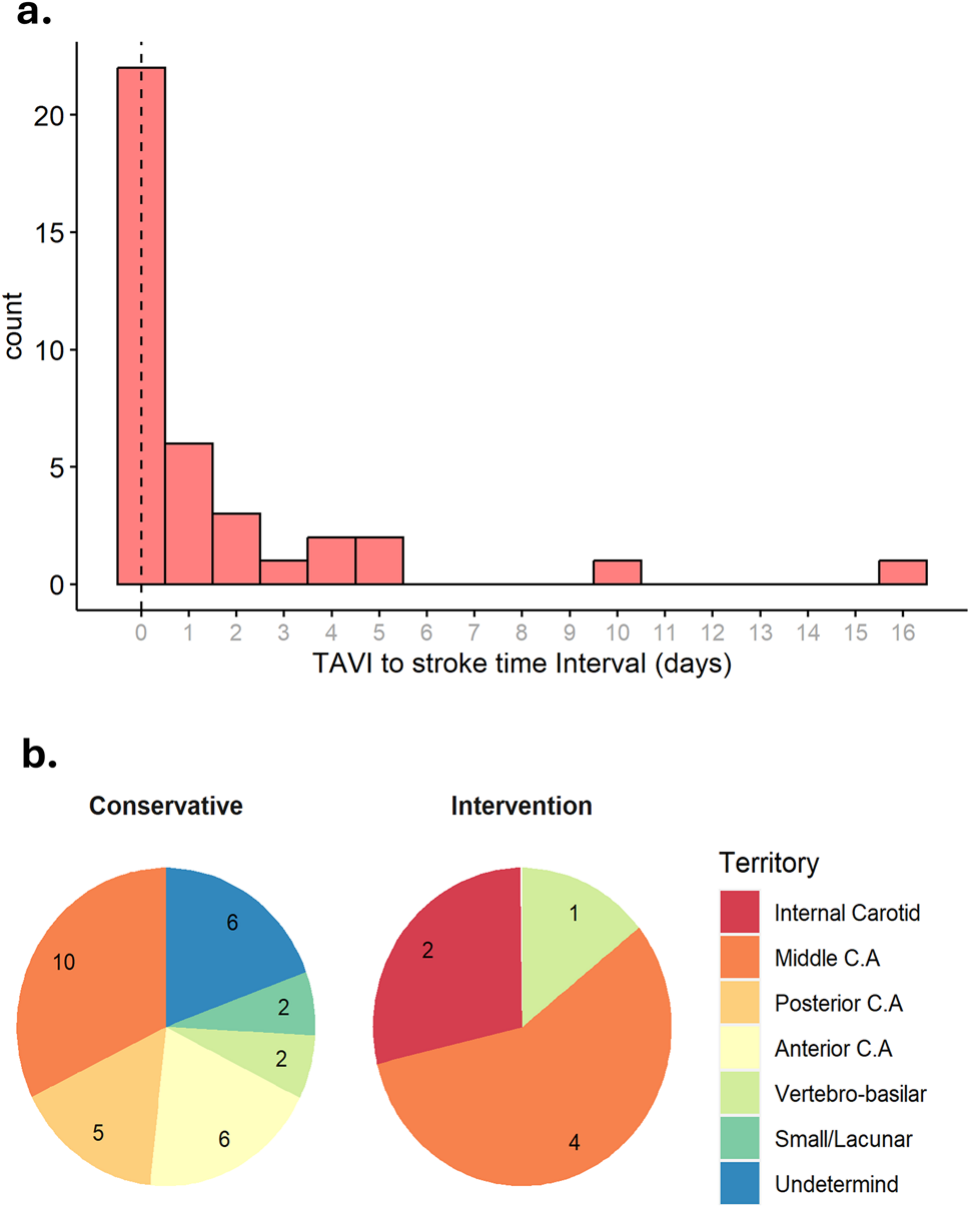
**a** TAVI to stroke time interval,** b** Stroke territory distributions

Of the 1515 consecutive patients who underwent TAVI between 2008 and 2021, 38 developed AISCT within 30 days (supplemental figure SI). TAVI to stroke time interval is presented in Fig. 1 (panel a). Most strokes were observed within the first 24 h following TAVI (*p* < 0.01). Baseline and procedural characteristics are shown in Table 1. Patients in the AISCT + group had higher rates of peripheral vascular disease (27.3% vs. 11.6%, *p* = 0.01), atrial fibrillation/flutter (58.3% vs. 28.7%, *p* < 0.01), and prior ischemic stroke (41.7% vs. 16.5%, *p* < 0.01), as compared to the AISCT- group. Other baseline characteristics were evenly distributed between groups. There were no significant differences in AS severity indices (i.e., aortic valve area, peak and mean trans-aortic pressure gradients) between the groups. In term of TAVI procedural characteristics, patients in the AISCT + group were less often treated via transfemoral access (80.6% vs. 95.3%, *p* < 0.01), as compared to the AISCT- group. Numerically higher rate of general anesthesia (GA) (22.2% vs. 11.0%, *p* = 0.06), balloon pre-dilatation (45.2% vs. 30.8%, *p* = 0.13), and a lower rate of balloon post-dilatations (13.8% vs. 26.8%, *p* = 0.17) were observed in the AISCT + group, as compared to the AISCT- group. Baseline and procedural characteristics of patients with AISCT (2008–2021) are presented in Table 2. Out of the 38 patients, 31 were treated conservatively, while the remaining 7 underwent NI [of which 6 underwent mechanical thrombectomy (MT), and one thrombolytic therapy (TT)]. There were no significant differences in age, gender, traditional risk factors, previous cardiac procedures, operative risk scores, functional class, AS severity, procedural access site, choice of anesthesia, use of embolic protection device, or type of valves between the two groups. The stroke characteristics are presented in Table 3. Mild stroke (NIHSS 1–5) constituted 52.6% (*n* = 20) of AISCT and were all treated conservatively. Moderate strokes (NIHSS 6–14) constituted 39.5% (*n* = 15), of which 10 (66.6%) were treated conservatively and 5 (33.3%) underwent NI. Three strokes were classified as severe (NIHSS > 14), of which 2 underwent NI. Stroke territory distributions between the two groups are presented in Fig. 1 (panel b).

**Table 1:** Baseline and procedural characteristics of patients after TAVI (2008–2021)

Variables	No stroke *N* = 1477 (%)	Stroke *N* = 38 (%)	*P* value
Age	80.5 ± 7.3	79.8 ± 15.8	0.79
Gender-F	761 (51.5)	17 (47.2)	0.73
BMI	28.2 ± 5.5	27.0 ± 5.1	0.18
Hemoglobin (g/dL)	12.0 ± 1.7	11.8 ± 1.7	0.73
Creatinine (g/dL)	1.2 ± 0.8	1.2 ± 0.7	0.85
Hypertension	1365 (95.5)	35 (97.2)	0.92
Diabetes Mellitus	655 (46.3)	18 (50.0)	0.79
PVD	157 (11.6)	9 (27.3)	0.01
Atrial fibrillation/flutter	390 (28.7)	21 (58.3)	< 0.01
Normal left ventricular function (> 55%)	1061 (71.8)	23 (63.9)	0.39
Prior ischemic stroke (CVA or TIA)	223 (16.5)	15 (41.7)	< 0.01
Prior myocardial infarction	165 (14.1)	5 (17.2)	0.83
Prior CABG	228 (19.4)	12 (33.3)	0.06
STS Score	4.5 ± 3.6	5.7 ± 4.4	0.11
Aortic peak pressure (mm/Hg)	75.0 ± 22.9	75.4 ± 18.2	0.89
Aortic mean pressure (mm/Hg)	47.5 ± 16.4	48.1 ± 12.4	0.78
Aortic valve area (cm^2^)	0.7 ± 0.2	0.6 ± 0.2	0.15
General anesthesia	162 (11.0)	8 (22.2)	0.06
Femoral access	1408 (95.3)	29 (80.6)	< 0.01
Balloon pre-dilatation	411 (30.8)	14 (45.2)	0.13
Balloon post-dilatation	349 (26.8)	4 (13.8)	0.17
Balloon expandable valve	433 (29.3)	9 (25.0)	0.20
Hospitalization length (days)	4.0 ± 3.1	7.7 ± 7.2	< 0.01

Values are mean ± SD or *n* (%)

*TAVI* transcatheter aortic valve implantation; *BMI* body mass index; *PVD* peripheral vascular disease; *CVA* cerebrovascular accident; *TIA* transient ischemic attack; *CABG* coronary artery bypass graft; *STS* society

**Table 2:** Baseline and procedural characteristics of patients with AISCT (2008–2021)

Variable	All Stroke N = 38 (%)	Conservative Therapy *N* = 31 (%)	Neurointervention *N* = 7 (%)	*P* value
Age	80.6 ± 15.6	80.0 ± 16.9	83.4 ± 7.6	0.42
Gender-M	18 (47.4)	13 (41.9)	5 (71.4)	0.22
Hemoglobin (g/dL)	11.8 ± 1.7	11.9 ± 1.6	11.5 ± 2.0	0.62
Creatinine (g/dL)	1.1 ± 0.7	1.2 ± 0.7	1.0 ± 0.3	0.30
Hypertension	31 (81.6)	27 (87.1)	4 (57.1)	0.10
Diabetes mellitus	19 (50.0)	16 (51.6)	3 (42.9)	N/S
COPD	9 (23.7)	8 (25.8)	1 (14.3)	N/S
Prior ischemic stroke (CVA or TIA)	15 (39.5)	12 (38.7)	3 (42.9)	N/S
Previous myocardial Injury	16 (42.1)	12 (38.7)	4 (57.1)	0.53
Previous PCI	27 (71.1)	24 (77.4)	3 (42.9)	0.19
Previous CABG	12 (31.6)	10 (32.3)	2 (28.6)	N/S
Previous Valve Surgery	0 (0.0)	0 (0.0)	0 (0.0)	N/S
Valve in Valve	1 (2.6)	0 (0.0)	1 (14.3)	N/S
STS Score	5.4 ± 4.2	5.7 ± 4.3	4.0 ± 3.5	0.28
EuroScore 2	5.6 ± 5.0	5.8 ± 5.3	4.5 ± 1.0	0.36
Atrial Fibrillation/Flutter	21 (55.3)	17 (54.8)	4 (57.1)	N/S
Pacemaker/ICD	4 (10.5)	4 (12.9)	0 (0.0)	N/S
Porcelain aorta	10 (35.7)	8 (34.8)	2 (40.0)	N/S
Baseline NYHA	3.1 ± 0.7	3.0 ± 0.8	3.2 ± 0.4	0.54
Frailty	18 (58.1)	15 (57.7)	3 (60.0)	N/S
Calcium Score	2,299 ± 908	2,418 ± 892	1,851 ± 953	0.34
Aortic peak pressure (mm/Hg)	74.0 ± 18.2	74.8 ± 19.1	70.4 ± 14.7	0.52
Aortic mean pressure (mm/Hg)	47.3 ± 12.4	47.5 ± 12.9	46.1 ± 10.6	0.77
Aortic valve area (cm^2^)	0.6 ± 0.2	0.6 ± 0.1	0.7 ± 0.3	0.60
Bicuspid aortic valve	1 (2.6)	1 (3.2)	0 (0.0)	N/S
Femoral access	32 (83.8)	25 (80.0)	7 (100.0)	0.57
General anesthesia	10 (26.3)	10 (32.3)	0 (0.0)	0.16
Embolic protective device	1 (2.6)	1 (3.2)	0 (0.0)	N/S
Self-expandable valve	24 (72.7)	19 (73.1)	5 (71.4)	N/S

Values are mean ± SD or *n* (%)

*AISCT* acute ischemic stroke complicating TAVI; *TAVI* transcatheter aortic valve implantation; *BMI* body mass index; *GFR* glomerular filtration rate; *PVD* peripheral vascular disease; *COPD* chronic obstructive pulmonary disease; *CVA* cerebrovascular accident; *TIA* transient ischemic attack; *PCI* percutaneous coronary intervention; *CABG* coronary artery bypass graft; *STS* society of thoracic surgeons; *EuroScore* European system for cardiac operative risk evaluation; *ICD*, implantable cardioverter defibrillator; *NYHA* New York Heart Association

**Table 3:** Stroke characteristics (2008–2021)

Variable	All Stroke *N* = 38 (%)	Conservative Therapy *N* = 31 (%)	Neurointervention *N* = 7(%)	*P* value
Mild stroke (NIHSS 1–5)	20 (52.6)	20 (64.5)	0 (0.0)	< 0.01
Moderate stroke (NIHSS 6–14)	15 (39.5)	10 (32.2)	5 (71.4)	N/S
Severe stroke (NIHSS > 15)	3 (7.9)	1 (3.2)	2 (28.6)	N/S
Median (95% CI) NIHSS Score	4 (2.75–8)	3 (2–6)	9 (8–14)	< 0.01
No disability (mRS 0–1)	13 (34.2)	12 (38.7)	1 (14.3)	N/S
Disability (mRS 2–5)	16 (42.1)	12 (38.7)	4 (57.1)	N/S
Unavailable mRS	5 (13.2)	5 (16.1)	0 (0.0)	N/S
Death resulting from stroke	4 (10.53)	2 (6.5)	2 (28.6)	N/S
Median (95% CI) mRS Score	3 (2–4.75)	3 (2–4)	5 (3–6)	< 0.01
Internal carotid artery	2 (5.3)	0 (0.0)	2 (28.6)	0.04
Middle cerebral artery	14 (36.8)	10 (32.3)	4 (57.1)	N/S
Posterior cerebral artery	5 (13.2)	5 (16.1)	0 (0.0)	N/S
Anterior cerebral artery	6 (15.8)	6 (19.4)	0 (0.0)	N/S
Vertebro-basilar artery	3 (7.9)	2 (6.5)	1 (14.3)	N/S
Small/lacunar artery	2 (5.3)	2 (6.5)	0 (0.0)	N/S
Undetermined	6 (15.8)	6 (19.4)	0 (0.0)	N/S

Values are mean ± SD or n (%)

*CT* computed tomography; *MRI* magnetic resonance imaging; *MRA* magnetic resonance angiography; *NIHSS* national institute of health stroke

### Clinical outcomes

**Fig. 2:**
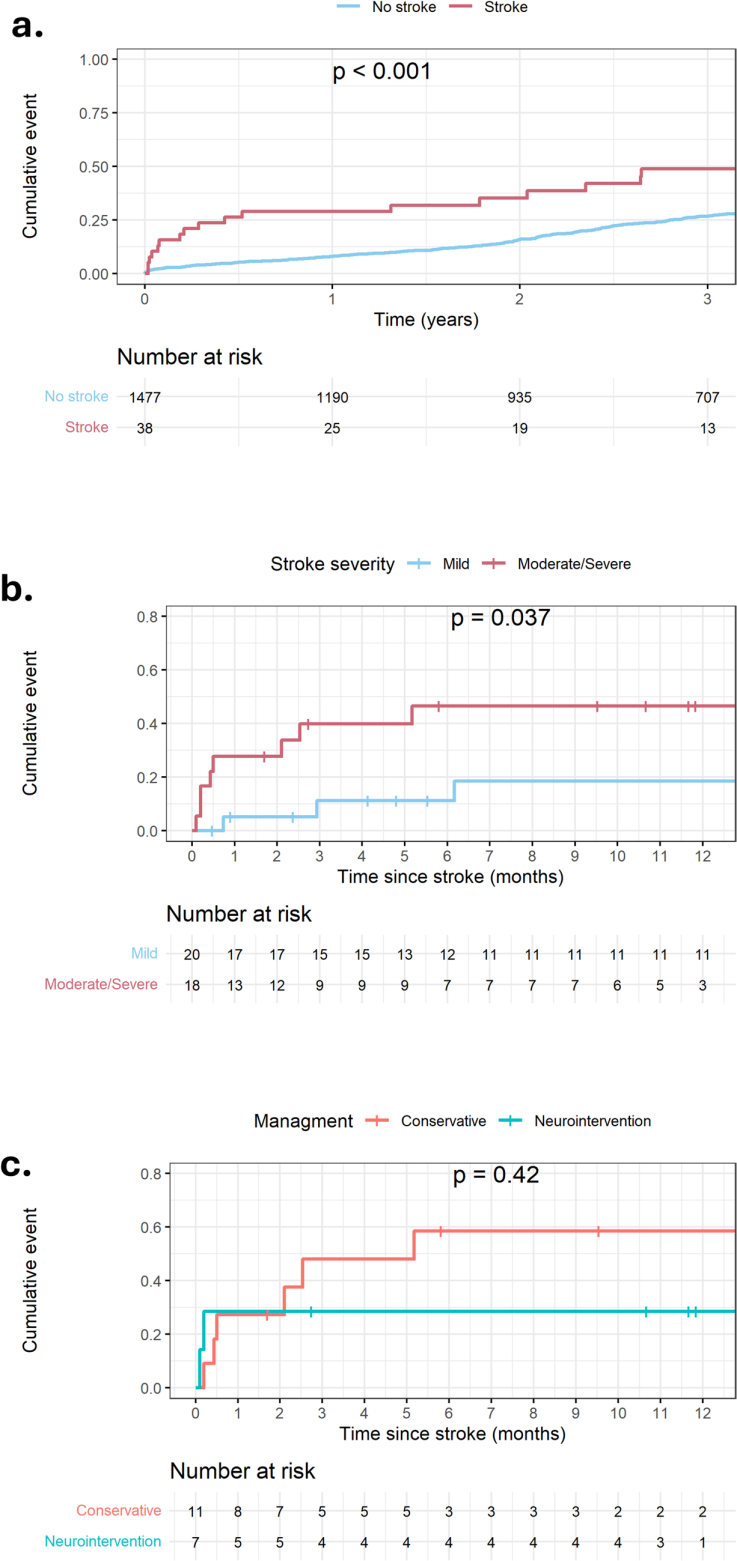
KM curves for mortality

KM curves for mortality are presented in Fig. 2. All-cause mortality was significantly higher in the AISCT group at 30 days (13.9% vs. 2.4%, *p* < 0.01), 1-year (27.8% vs. 8.1%, *p* < 0.01), and 3 years (49.0% vs. 26.8%, *p* < 0.01), as compared to the AISCT-group (panel a). There were no significant differences in rates of new pacemaker implantations, or vascular complications between the two groups (Table 4). Results of the univariate and multivariate logistic regression analysis for AISCT are shown in supplemental figures S2 and S3 (respectively). Prior ischemic stroke (*p* < 0.01), atrial fibrillation/flutter (*p* < 0.01), PVD (*p* < 0.01), GA (*p* = 0.04), and non-femoral access (*p* < 0.01) were associated with an increased risk of AISCT. In a model incorporating the aforementioned covariates, pervious CVA (OR = 3.5; 95% CI: 1.68–7.34, *p* < 0.001), atrial fibrillation/flutter (OR = 3.17, 95% CI 1.56–6.59, *p* < 0.001), and non-femoral access (OR = 5.71, 95% CI 1.20–34.31, *p* = 0.04) emerged as independent predictors of AISCT. KM curves for 1-year mortality following stroke, grouped by severity, are presented in Fig. 2 (panel b). All-cause mortality following moderate/severe stroke was significantly higher (*p* = 0.037) compared to mild stroke.

**Table 4:** Clinical outcomes during follow-up (2008–2021)

Variables	No stroke *N* = 1477 (%)	Stroke *N* = 38 (%)	*P* value
New pacemaker implantation	195 (14.8)	8 (25.0)	0.18
Major vascular complication	35 (2.4)	1 (2.8)	1.00
All-cause mortality at 30 days	35 (2.4)	5 (13.9)	< 0.01
All-cause mortality at 1 year	120 (8.1)	10 (27.8)	< 0.01
All-cause mortality at 3 years	396 (26.8)	18 (49.0)	< 0.01

Values are mean ± SD or *n* (%)

### Conservative approach compared to neurointervention

**Fig. 3:**
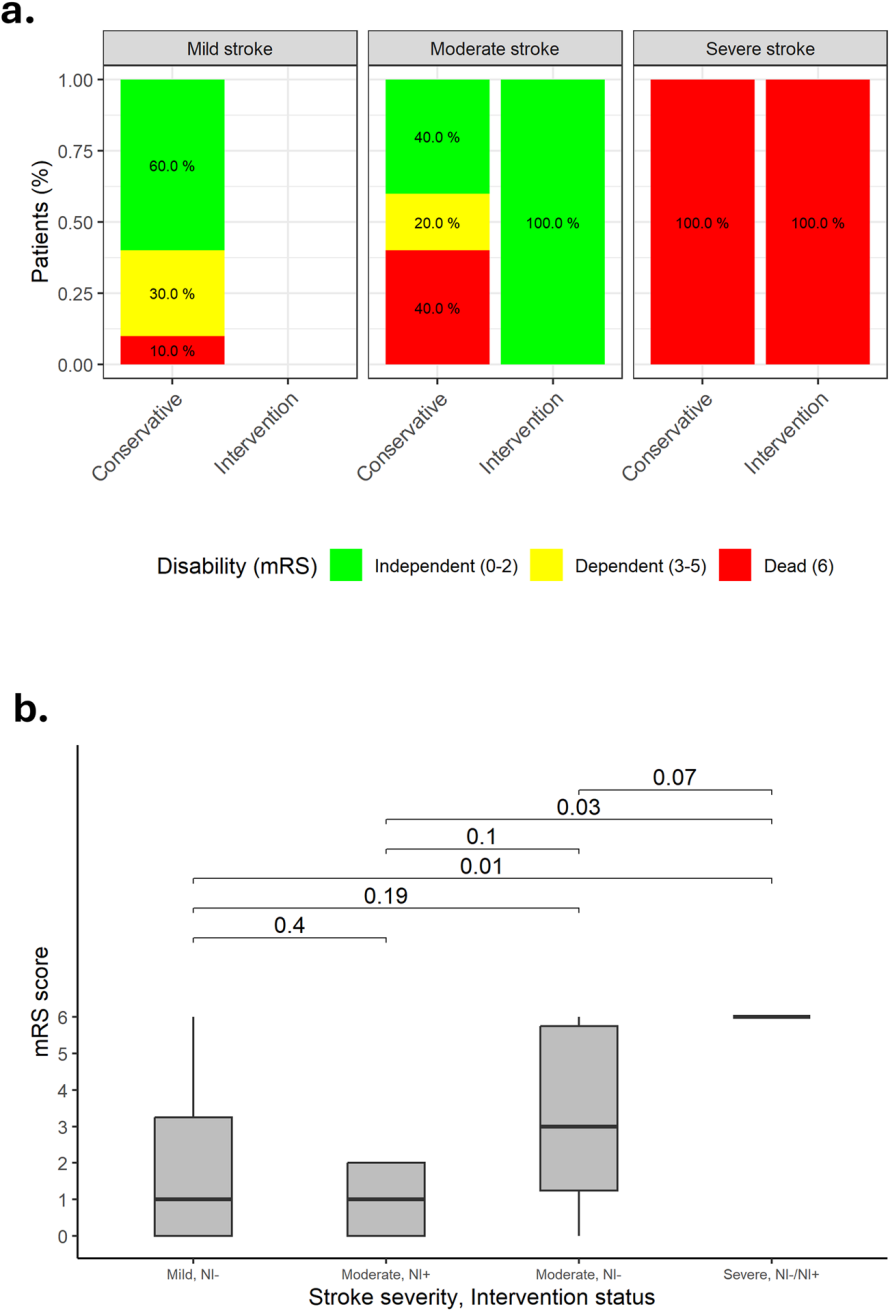
Neurologic disability status at 90 days

**Fig. 4:**
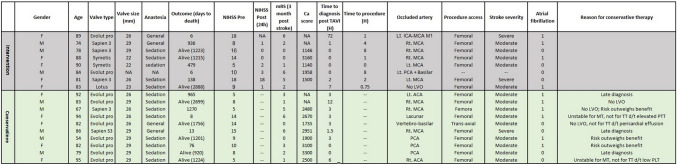
Detailed descriptions of all patients with moderate or severe stroke who underwent NI or were treated conservatively

In patients who were considered potential candidates for NI (i.e. moderate or severe stroke; *n* = 18), a CA was compared to NI. The clinical outcomes are presented in Table 5. There were no significant differences in the in-hospital, 30 days and 1-year mortality between the two groups (Fig. 2, panel c). Neurologic disability status at 90 days is presented in Fig. 3. Median mRS at 3 months was 1 (IQR 0–3.25) following mild stroke, 1 (IQR 0–2) following moderate stroke and NI, 3 (IQR 1.25–5.75) following moderate stroke without NI, and 6 (IQR 6–6) following severe stroke, regardless of NI. Following moderate stroke, patients undergoing NI enjoyed a higher rate of disability free survival at 3 months (100% compared to 40% respectively, *p* = 0.044), with a non-significantly lower median mRS as compared to CM (*p* = 0.1). There were no significant differences in in-hospital bleeding, vascular complications, or rates of new pacemaker implantations between the two groups. The detailed descriptions of all patients with moderate or severe stroke who underwent NI or were treated conservatively, are presented in Fig. 4. Most patients who underwent NI were diagnosed either during the TAVI procedure, right after TAVI completion, or as soon as the patient reached the intensive cardiac care unit. All MT were preformed to treat occlusions involving the middle cerebral artery (*n* = 4) or the internal carotid artery and middle cerebral artery (*n* = 2) segments, excluding one intervention preformed to treat the basilar to left posterior cerebral artery segments. NIHSS score of patients with AISCT before and after NI is presented in supplemental figure S4. Six out of seven interventions were successful in achieving a significant reduction in stoke severity. Following NI, median NIHSS has declined from 9 (IQR 7–14.5) to 1 (IQR 0.5–2.5), (*p* = 0.036).

**Table 5:** Clinical Outcomes: Conservative Therapy vs. Neurointervention in Patients with Moderate or Severe Stroke (NHISS > 5) (2008–2021)

Variable	Conservative Treatment *N* = 11 (%)	Neurointervention *N* = 7 (%)	*P* value
In-Hospital Bleeding	4 (36.3)	3 (42.8)	N/S
Acute Kidney Injury	5 (45.5)	0 (0.0)	0.04
Vascular Complications	4 (36.3)	3 (42.8)	N/S
New Pacemaker Implantation	1 (9.1)	1 (14.3)	N/S
No Disability (mRS 0–1) at 3 months	4 (36.4)	5 (71.4)	N/S
Disability (mRS 2–5) at 3 months	0 (0.0)	0 (0.0)	N/S
Death Resulting from Stroke at 3 months	7 (63.6)	2 (28.6)	N/S
In-Hospital Mortality	3 (27.3)	2 (28.6)	N/S
All-Cause Mortality at 30 days	3 (27.3)	2 (28.6)	N/S
All-Cause Mortality at 1-year	6 (58.4)	2 (28.6)	N/S

Values are mean ± SD or *n* (%)

*NIHSS* national institute of health stroke; *mRS* modified Rankin scale

**Fig. 5:**
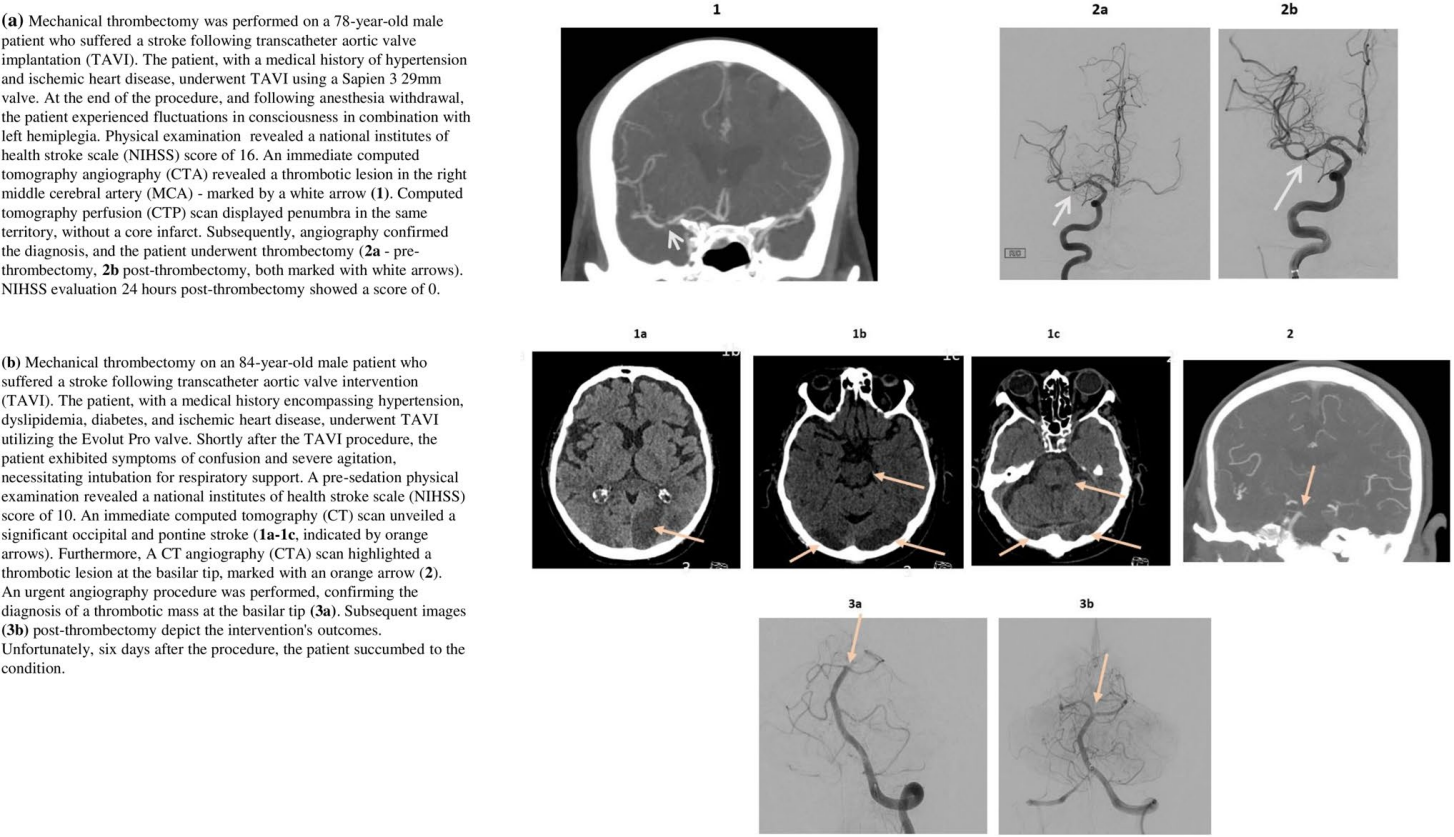
Exemplary cases of patients who underwent NI

Common reasons for not performing NI were the absence of a large vessel occlusion seen in brain CTA (*n* = 5), late diagnosis (*n* = 3), or perceived high risk of adverse outcomes / procedural failure (*n* = 3). Exemplary cases of patients who underwent NI are presented in Fig. 5.

## Discussion

In this registry-based study, which included 1515 consecutive TAVI procedures performed between 2008 and 2021, we have identified 38 (2.5%) AISCT events within 30 days. Most AISCT occurred within the first 24 h of the procedure (57.8%). A detailed analysis of these patients revealed that approximately half had mild stroke and fewer than 10% had a severe stroke. AISCT was associated with a fourfold increase in 1-year mortality (*p* < 0.01). As compared to mild stroke, moderate stroke or worse was associated with a 3.7-fold increase in mortality (*p* = 0.038). Prior ischemic stroke, atrial fibrillation/flutter, PVD, GA, and non-femoral access emerged as risk factors for AISCT. Among 18 patients who were considered candidates for NI (moderate or severe stroke), 6 underwent attempted MT and one patient was given TT with a median NIHSS decline from 9 to 1 (*p* = 0.036). As compared to the CM, NI was not found to reduce mortality. However, patients with moderate severity stroke (NIHSS score 6–14) who underwent NI, enjoyed a higher rate of disability free survival at 3 months compared to patients treated conservatively (*p* = 0.044).

Contemporary studies have consistently demonstrated 30-day AISCT rates approximating 2–3% [9, 10]. Given that: (a) stroke is associated with a significant increase in mortality, (b) roughly 50% of AISCT could be considered moderate or severe and are potentially disabling strokes, (c) AISCT is a largely stochastic event with no available high-quality models or risk scores to predict, (d) patients consider stroke a worse outcomes than death [11], and that (e) despite the extensive experience with cerebral embolic protection devices (CEPDs) no strategy has been shown to consistently prevent AISCT [12–14], AISCT remains a concerning issue in the current TAVI practice.

The management of AISCT is challenging. In this regard, we have previously published data from a large multicenter cohort of 39 NI (13 TT, 26 attempted MT) in 387 AISCT events (The ASTRO-TAVI registry [15]), showing that NI may be beneficial in increasing rates of independent survival at 90 days (from 37.5 to 60% in moderate severity stroke, *p* = 0.016). We have thus advocated for the formation of institutional rapid response heart-brain teams to enable a timely response to AISCT and to facilitate NI when appropriate in a timely manner. Interestingly, in the ASTRO-TAVI, only approximately 25% of the patients with moderate or severe stroke underwent NI, of which 33% were given TT, potentially putting patients in an increased hazard of bleeding and vascular complications given recent large bore vascular access, in addition to an increased risk of cerebral hemorrhages given the typical characteristics of TAVI patients in combination with frequent blood pressure fluctuations. This was largely affected by a lack of interventional neuroangiograhpy capabilities in some of the participating centers.

To further explore dilemmas and strategies in ASICT management, in the current analysis we have studied our singular institutional experience of AISCT in a higher granularity. Rabin medical center is a tertiary hospital with an onsite neurology consultant and a dedicated neurological catheterization laboratory, which is accessible anytime to facilitate urgent and elective procedures. When a stroke code is activated post TAVI, a rapid neurological evaluation is performed followed by a brain CTA and a CT perfusion, if needed. Several scenarios can than unfold—in the presence of a moderate or severe stroke (NIHSS > 5) and a large vessel occlusion (LVO), the patient is considered for MT. In the presence of moderate or severe stroke and no LVO, the patient is considered for TT, if the bleeding risk is not prohibitive. In the presence of mild stroke (NIHSS ≤ 5), or in the combination of moderate or severe stroke, no LVO and prohibitive bleeding risk, the patient is managed with a CM.

In our local experience, of 18 patients with moderate or severe stroke only 7 underwent NI (39%). Of the remaining 11, three were denied therapy due to late diagnosis with little or no remaining penumbra at CT perfusion, five were denied due to the absence of LVO or a difficult to approach occlusion (PCA) and were not offered TT given a perceived high bleeding risk, and three were considered at high risk for any intervention.

Despite the small number of patients treated, several of our observations support the notion that meticulously selective NI to treat AISCT may be both safe and effective—(1) median NIHSS has declined dramatically after NI, (2) independent survival rate (mRS 0–2) at 3 months was significantly higher in NI as compared to CM in the moderate severity group, (3) median mRS score following NI for moderate severity stroke (median 1, IQR 0–2) resembled median mRS after mild stroke (median 1, IQR 0–3.25), and was numerically lower than mRS after CM for moderate severity stroke (median 3 IQR 1.25–5.75, *p* = 0.1).

Regarding the choice of intervention, as compared to MT, TT may be associated with both lower safety and reduced efficacy. Previous data suggest that TT after AISCT may be associated with an increased risk of major access site bleeding [15, 16]. In terms of treatment efficacy, considering that tissue-derived debris is often found in the assessment of embolized material collected from CEPD post TAVI [17, 18], MT targeting the retrieval of embolized material should be preferred over TT. TT should be considered in the absence of LVO or when access to MT is limited. Since only one patient in our cohort was treated with TT, we cannot offer any insights regarding the effectiveness of this strategy after AISCT.

As reflected in our observation, timely diagnosis of AISCT is a major determinant of effective treatment. In this regard, several strategies may facilitate earlier diagnosis of AISCT. The first is adherence to conscious sedation (CS) rather than to GA during TAVI. While GA has not been identified as an independent predictor of AISCT [19], it may lead to an earlier diagnosis, occasionally while the patient is still on the catheterization table, and in turn to a rapid initiation of the diagnosis-imaging-treatment cascade. Furthermore, in the ASTRO-TAVI registry [15], GA was significantly more prevalent in the CM as compared to the NI group (14% compared to 38% respectively, *p* = 0.01), possibly implying that a rapid diagnosis of AISCT is linked to higher rates of intervention. In the current analysis, 4/7 patients in the NI groups were diagnosed with AISCT during or immediately after TAVI, all of whom underwent TAVI under CS. Of particular interest, is a case in which a 90-year-old female, who developed clear focal neurologic stigmata immediately after TAVI, underwent neuro angiography while still in the cardiac catheterization lab. Thrombus was aspirated from the left middle cerebral artery (MCA) with a good angiographic result. The symptoms improved rapidly, and the patient recovered with no significant disability.

The second factor in the chain of AISCT diagnosis is the availability of systematic evaluation by a neurologist. Recent studies have demonstrated that the detection of overt and covert CNS injury is directly related to the intensity of surveillance, with systematic examination by neurologists and routine CNS imaging yielding substantially higher event rates [20]. Furthermore, reported AISCT rates were found to be significantly higher in comprehensive stroke centers (CSC), as compared to non-CSC designated centers, suggesting that higher skills in stroke treatment and detection may promote higher rate of AISCT diagnosis [21]. It is therefore imperative to have a low threshold to assess all TAVI patients with a timely neurologic assessment, and to perform the necessary imaging when needed. Assuming 1.5% rate of 30 moderate/severe AISCT with a NI eligibility of about 50%, one patient in every 133 TAVI cases could benefit from a NI after an AISCT.

### Limitations

Our study has several limitations: first, despite the absence of significant differences between the groups, there is a possible selection bias to CM in sicker patients. Indeed, three patients were deemed unsuitable for any intervention, all ending up with an unfavorable neurologic outcome. Second, some relevant data fields were not collected in our registry including CEPD use (low rate, approximately 5%), bicuspid aortic valve, and calcium score. Finally, the low number of NI may lead to a type 2 error in the interpretation of therapy related outcome and should be therefore considered as hypothesis generating.

## Conclusions

AISCT is associated with increased morbidity and mortality, which is highly impacted by stroke severity. Prompt diagnosis and treatment are crucial for achieving optimal neurologic outcome and reducing mortality. Our findings suggest possible disability improvement in patients with moderate stroke, following timely NI. Additional research is required to support this hypothesis.

## Supplementary Information

Below is the link to the electronic supplementary material.

Supplementary file1 (TIFF 6328 KB)

Supplementary file2 (TIFF 14238 KB)

Supplementary file3 (TIFF 6566 KB)

Supplementary file4 (TIFF 6328 KB)

## Data Availability

The data underlying this article will be shared on reasonable request to the corresponding author.

## References

[1] Vahanian A, Beyersdorf F, Praz F, et al. 2021 ESC/EACTS Guidelines for the management of valvular heart disease: developed by the Task Force for the management of valvular heart disease of the European society of cardiology (ESC) and the European association for cardio-thoracic surgery (EACTS). Rev Esp Cardiol (Engl Ed). 2022;75(6):524. 10.1016/j.rec.2022.05.006. 35636831 10.1016/j.rec.2022.05.006

[2] Tamburino C, Valvo R, Criscione E, et al. The path of transcatheter aortic valve implantation: from compassionate to low-risk cases. Eur Heart J Suppl. 2020;22(Supplement_L):L140–5. 10.1093/eurheartj/suaa154. 33239989 10.1093/eurheartj/suaa154 PMC7673604

[3] Carroll JD, Mack MJ, Vemulapalli S, et al. STS-ACC TVT registry of transcatheter aortic valve replacement. Ann Thorac Surg. 2021;111(2):701–22. 10.1016/j.athoracsur.2020.09.002. 33213826 10.1016/j.athoracsur.2020.09.002

[4] Castelo A, Grazina A, Teixeira B, et al. Outcomes and predictors of periprocedural stroke after transcatheter aortic valve implantation. J Stroke Cerebrovasc Dis. 2023;32(5): 107054. 10.1016/j.jstrokecerebrovasdis.2023.107054. 36881984 10.1016/j.jstrokecerebrovasdis.2023.107054

[5] Maier O, Bosbach G, Piayda K, et al. Cerebrovascular events after transcatheter aortic valve replacement: the difficulty in predicting the unpredictable. JCM. 2022;11(13):3902. 10.3390/jcm11133902. 35807187 10.3390/jcm11133902 PMC9267500

[6] Goyal M, Menon BK, van Zwam WH, et al. Endovascular thrombectomy after large-vessel ischaemic stroke: a meta-analysis of individual patient data from five randomised trials. The Lancet. 2016;387(10029):1723–31. 10.1016/S0140-6736(16)00163-X. 10.1016/S0140-6736(16)00163-X 26898852

[7] Généreux P, Piazza N, Alu MC, et al. Valve academic research consortium 3: updated endpoint definitions for aortic valve clinical research. J Am Coll Cardiol. 2021;77(21):2717–46. 10.1016/j.jacc.2021.02.038. 33888385 10.1016/j.jacc.2021.02.038

[8] Brott T, Adams HP, Olinger CP, et al. Measurements of acute cerebral infarction: a clinical examination scale. Stroke. 1989;20(7):864–70. 10.1161/01.STR.20.7.864. 2749846 10.1161/01.str.20.7.864

[9] Almarzooq ZA, Kazi DK, Wang Y, et al. Outcomes of stroke events during transcatheter aortic valve implantation. EuroIntervention. 2022;18(4):e335–44. 10.4244/EIJ-D-21-00951. 35135749 10.4244/EIJ-D-21-00951 PMC10259241

[10] Davlouros PA, Mplani VC, Koniari I, et al. Transcatheter aortic valve replacement and stroke: a comprehensive review. J Geriatr Cardiol. 2018;15(1):95–104. 10.11909/j.issn.1671-5411.2018.01.008. 29434631 10.11909/j.issn.1671-5411.2018.01.008 PMC5803543

[11] Ahmad Y, Nijjer S, Cook CM, et al. A new method of applying randomised control study data to the individual patient: a novel quantitative patient-centred approach to interpreting composite end points. Int J Cardiol. 2015;195:216–24. 10.1016/j.ijcard.2015.05.109. 26048380 10.1016/j.ijcard.2015.05.109

[12] Kapadia SR, Makkar R, Leon M, et al. Cerebral embolic protection during transcatheter aortic-valve replacement. N Engl J Med. 2022;387(14):1253–63. 10.1056/NEJMoa2204961. 36121045 10.1056/NEJMoa2204961

[13] Reddy RK, Ahmad Y, Arnold AD, Howard JP. Cerebral embolic protection devices during transcatheter aortic valve replacement: a meta-analysis of randomized controlled trials. J Soc Cardiovasc Angiogr Interv. 2023;2(5): 101031. 10.1016/j.jscai.2023.101031. 37780935 10.1016/j.jscai.2023.101031 PMC10533415

[14] Suhai FI, Varga A, Szilveszter B, et al. Predictors and neurological consequences of periprocedural cerebrovascular events following transcatheter aortic valve implantation with self-expanding valves. Front Cardiovasc Med. 2022;9: 951943. 10.3389/fcvm.2022.951943. 36277778 10.3389/fcvm.2022.951943 PMC9581280

[15] Levi A, Linder M, Seiffert M, et al. Management and outcome of acute ischemic stroke complicating transcatheter aortic valve replacement. JACC: Cardiovasc Interv. 2022;15(18):1808–19. 10.1016/j.jcin.2022.06.033. 36137683 10.1016/j.jcin.2022.06.033

[16] Khera S, Koshy AN, Tang GHL, et al. Prevention and management of stroke after transcatheter aortic valve replacement: the Mount Sinai stroke initiative. JAHA. 2023;12(4): e028182. 10.1161/JAHA.122.028182. 36752233 10.1161/JAHA.122.028182 PMC10111471

[17] Danenberg H, Vaknin-Assa H, Makkar R, et al. First-in-human study of the CAPTIS embolic protection system during transcatheter aortic valve replacement. EuroIntervention. 2023;19(11):e948–52. 10.4244/EIJ-D-23-00465. 37916296 10.4244/EIJ-D-23-00465 PMC10719740

[18] Van Mieghem NM, Schipper MEI, Ladich E, et al. Histopathology of embolic debris captured during transcatheter aortic valve replacement. Circulation. 2013;127(22):2194–201. 10.1161/CIRCULATIONAHA.112.001091. 23652860 10.1161/CIRCULATIONAHA.112.001091

[19] Thiele H, Kurz T, Feistritzer HJ, et al. General versus local anesthesia with conscious sedation in transcatheter aortic valve implantation: the randomized SOLVE-TAVI trial. Circulation. 2020;142(15):1437–47. 10.1161/CIRCULATIONAHA.120.046451. 32819145 10.1161/CIRCULATIONAHA.120.046451

[20] Messé SR, Acker MA, Kasner SE, et al. Stroke after aortic valve surgery: results from a prospective cohort. Circulation. 2014;129(22):2253–61. 10.1161/CIRCULATIONAHA.113.005084. 24690611 10.1161/CIRCULATIONAHA.113.005084 PMC4043861

[21] Grossman PM, Sukul D, Lall SC, et al. The relationship between hospital stroke center designation and TVT reported stroke. JACC Cardiovasc Interv. 2023;16(2):168–76. 10.1016/j.jcin.2022.10.020. 36697152 10.1016/j.jcin.2022.10.020

