# Supplementary figures and images for: When brain and heart collide: a deeper dive into treatment pathways of stroke complicating TAVI
Source: Cardiovasc Interv Ther. 2025 Mar 29;40(3):657–68. doi: 10.1007/s12928-025-01121-w (PMC12167299; doi:10.1007/s12928-025-01121-w)

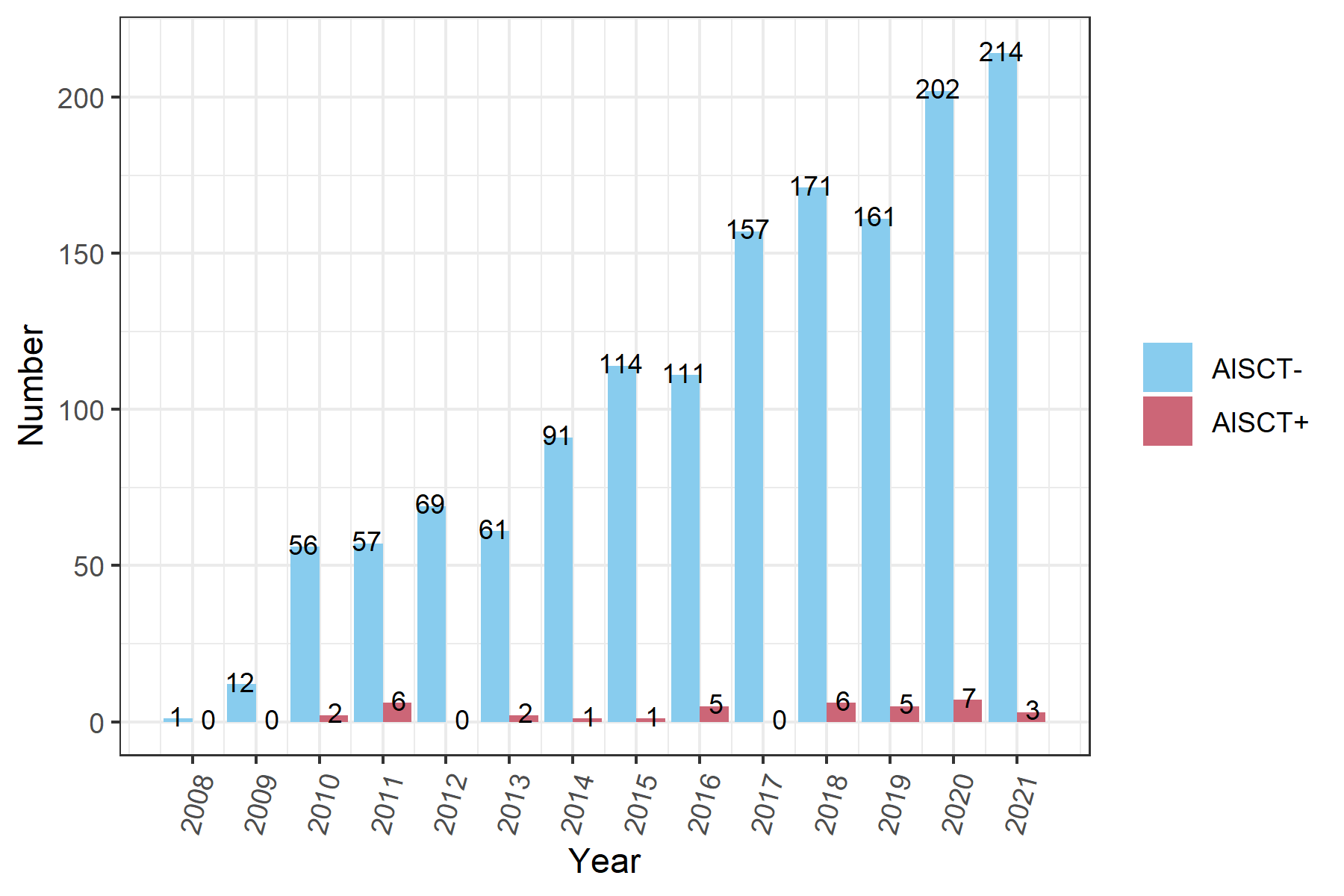

Supplement: Supplementary file 1 — Supplementary file1 (TIFF 6328 KB) [file 12928_2025_1121_MOESM1_ESM.tiff]

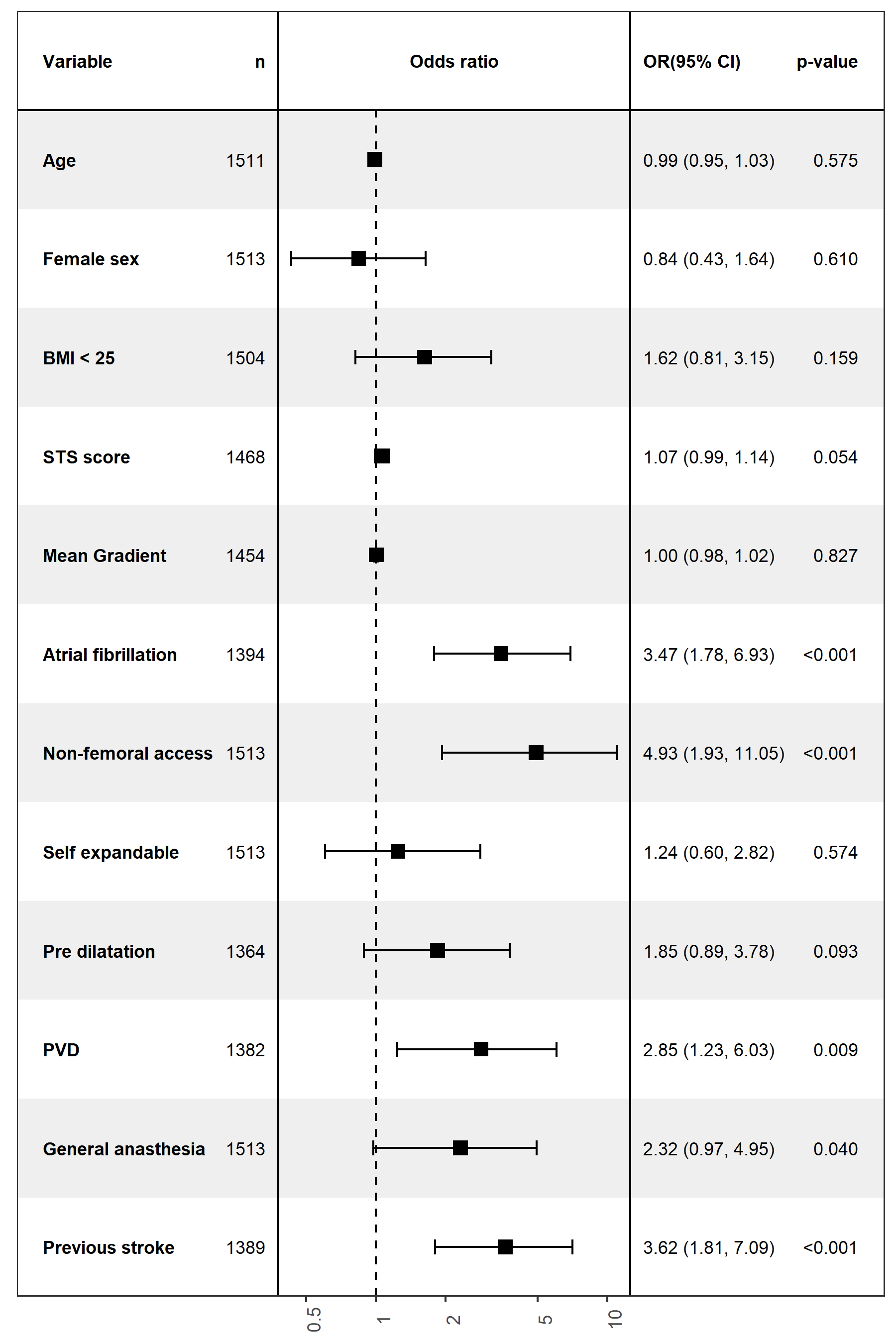

Supplement: Supplementary file 2 — Supplementary file2 (TIFF 14238 KB) [file 12928_2025_1121_MOESM2_ESM.tiff]

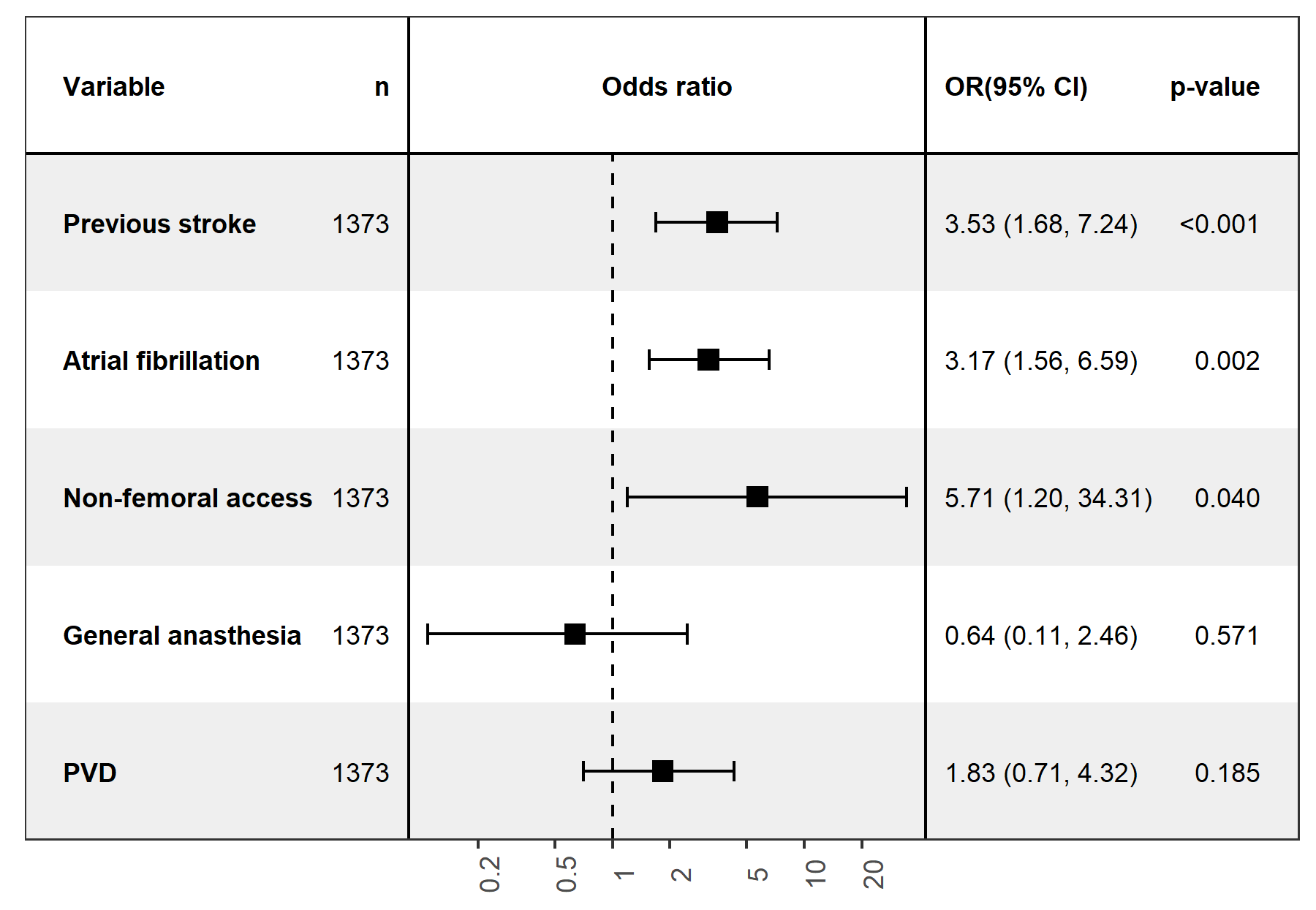

Supplement: Supplementary file 3 — Supplementary file3 (TIFF 6566 KB) [file 12928_2025_1121_MOESM3_ESM.tiff]

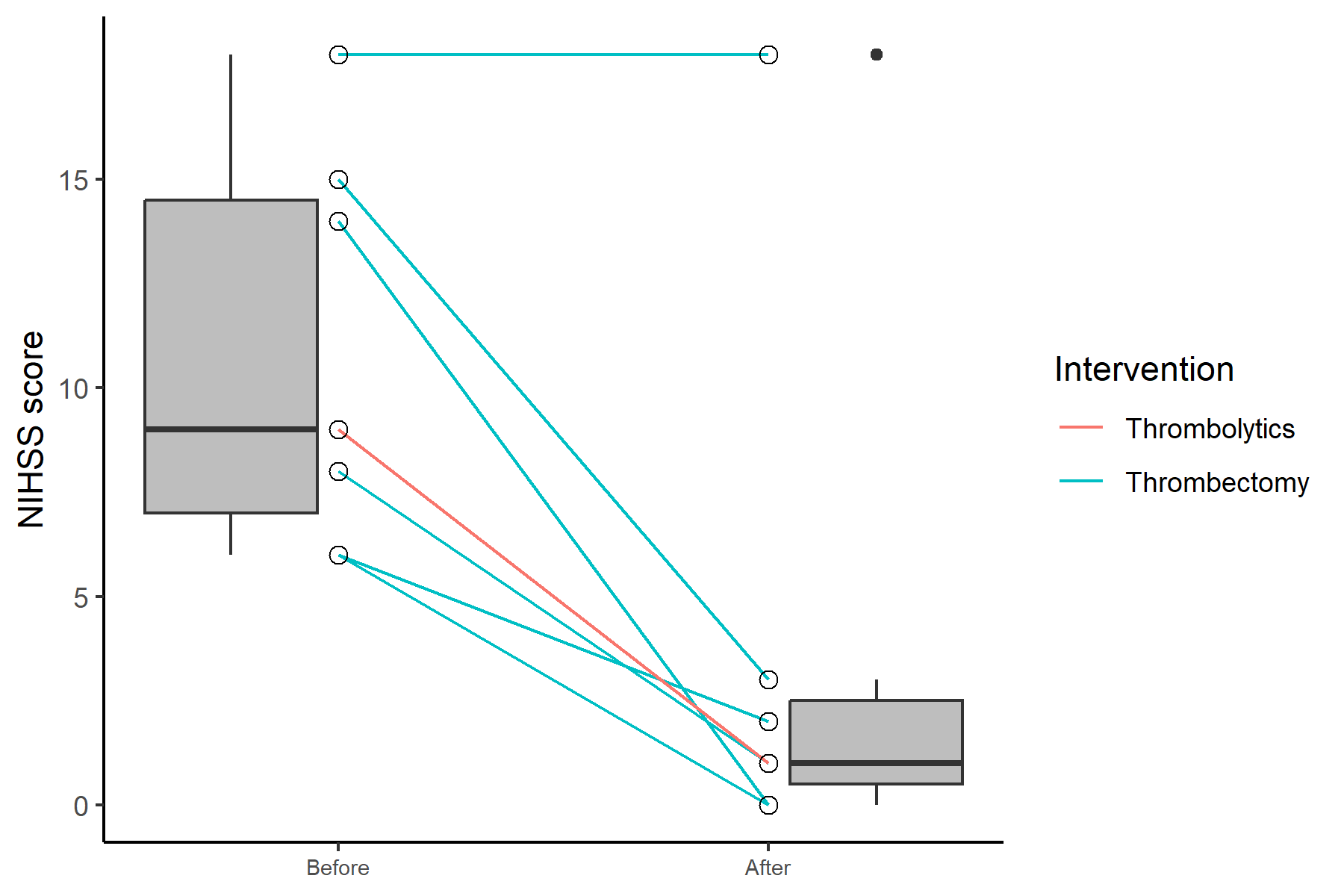

Supplement: Supplementary file 4 — Supplementary file4 (TIFF 6328 KB) [file 12928_2025_1121_MOESM4_ESM.tiff]
